# Quantitative differences in developmental profiles of spontaneous activity in cortical and hippocampal cultures

**DOI:** 10.1186/s13064-014-0028-0

**Published:** 2015-01-28

**Authors:** Paul Charlesworth, Ellese Cotterill, Andrew Morton, Seth GN Grant, Stephen J Eglen

**Affiliations:** Genes to Cognition Programme, Wellcome Trust Sanger Institute, Genome Campus, CB10 1SA Hinxton, UK; Current address: Department of Physiology, Development and Neuroscience, Physiological Laboratory, Downing Street, Cambridge, CB2 3EG, UK; Cambridge Computational Biology Institute, University of Cambridge, Wilberforce Road, Cambridge, CB3 0WA, UK; Current address: Centre for Integrative Physiology, University of Edinburgh School of Biomedical Sciences, EH8 9XD Edinburgh, UK; Centre for Clinical Brain Sciences and Centre for Neuroregeneration, Chancellors Building, Edinburgh University, 49 Little France Crescent, Edinburgh, EH16 4SB, UK

**Keywords:** Multielectrode array, Spontaneous activity, Cortex, Hippocampus, Principal component analysis, Support vector machine, Classification tree

## Abstract

**Background:**

Neural circuits can spontaneously generate complex spatiotemporal firing patterns during development. This spontaneous activity is thought to help guide development of the nervous system. In this study, we had two aims. First, to characterise the changes in spontaneous activity in cultures of developing networks of either hippocampal or cortical neurons dissociated from mouse. Second, to assess whether there are any functional differences in the patterns of activity in hippocampal and cortical networks.

**Results:**

We used multielectrode arrays to record the development of spontaneous activity in cultured networks of either hippocampal or cortical neurons every 2 or 3 days for the first month after plating. Within a few days of culturing, networks exhibited spontaneous activity. This activity strengthened and then stabilised typically around 21 days *in vitro*. We quantified the activity patterns in hippocampal and cortical networks using 11 features. Three out of 11 features showed striking differences in activity between hippocampal and cortical networks: (1) interburst intervals are less variable in spike trains from hippocampal cultures; (2) hippocampal networks have higher correlations and (3) hippocampal networks generate more robust theta-bursting patterns. Machine-learning techniques confirmed that these differences in patterning are sufficient to classify recordings reliably at any given age as either hippocampal or cortical networks.

**Conclusions:**

Although cultured networks of hippocampal and cortical networks both generate spontaneous activity that changes over time, at any given time we can reliably detect differences in the activity patterns. We anticipate that this quantitative framework could have applications in many areas, including neurotoxicity testing and for characterising the phenotype of different mutant mice. All code and data relating to this report are freely available for others to use.

## Background

During development, many parts of the nervous system generate patterns of spontaneous activity. These patterns of activity are thought to be instructive in the assembly of neural connectivity, for example by driving activity-dependent mechanisms [1]. To date, most recordings of spontaneous activity have been *in vitro*, although spontaneous activity *in vivo* has also been reported in hippocampus and in several cortical areas [2-6]. *In vitro* recordings are typically made with multielectrode arrays (MEAs), which contain at least 60 electrodes. These recordings allow us to assess activity at a range of levels from the single unit to the network. Beyond their relevance for understanding how activity might guide development of the nervous system, spontaneous activity recordings have also been used as an assay for network performance in applied settings, like neurotoxicity screening [7].

In recent years there has been significant interest in measuring the developmental patterns of spontaneous activity in networks cultured from neurons in control and experimental conditions [7-11]. Although many properties of spontaneous activity have been reported, we do not yet have a systematic sense of how these features change across development, or which features of neural activity are useful for describing the observed patterns of activity.

To address both these questions, we have cultured two types of network on MEAs and recorded their activity every 2 to 3 days up to around 1 month post-plating of neurons onto the array. In the first type of network, we cultured hippocampal neurons taken from embryonic mice. The second type of network was created using exactly the same protocol except with neurons dissected from cortex. Recordings of spontaneous activity from both types of network were quantified using 11 different features at the level of individual electrodes, pairs of electrodes or the entire array. We found that hippocampal networks tend to generate more regular bursting activity, including theta bursts, and more correlated activity than the corresponding cortical networks at the same age.

## Results

### Development of spontaneous activity

**Figure 1:**
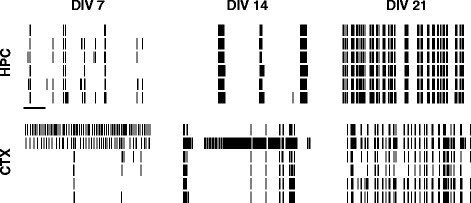
**Examples of spontaneous activity in developing cultures.** Top row: Hippocampal (HPC) cultures. Bottom row: Cortical (CTX) cultures. Each column represents one day *in vitro* (DIV). Within each raster plot, one row represents the spike train from one electrode; six (out of typically 59) electrodes are shown. Scale bar for all rasters is 10 s. CTX, cortex; DIV, days *in vitro*; HPC, hippocampus.

**Figure 2:**
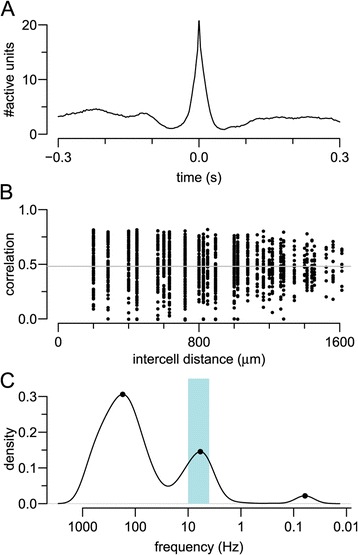
**Examples of features calculated for each recording.** The hippocampal recording from 14 DIV in Figure 1 was used as an example for this figure. **(A)** Mean network spike. **(B)** Pairwise correlation calculated using the spike time tiling coefficient. As there is weak dependence on distance, we take the mean (grey solid line). **(C)** Detection of theta bursting on an electrode with a firing rate close to the median activity on the array. DIV, days *in vitro*.

Within 7 days of culturing neurons on MEAs, spontaneous activity can be reliably recorded (Figure 1) from both hippocampal and cortical networks. As development progresses, we find that the firing rate increases, and that the frequency of bursting increases. To quantify these differences, we have used a range of measures (Figure 2) to assess the activity at a single-electrode level, pairwise and at the level of the entire network. All of these measures are defined in the methods.

#### Overall firing rates

**Figure 3:**
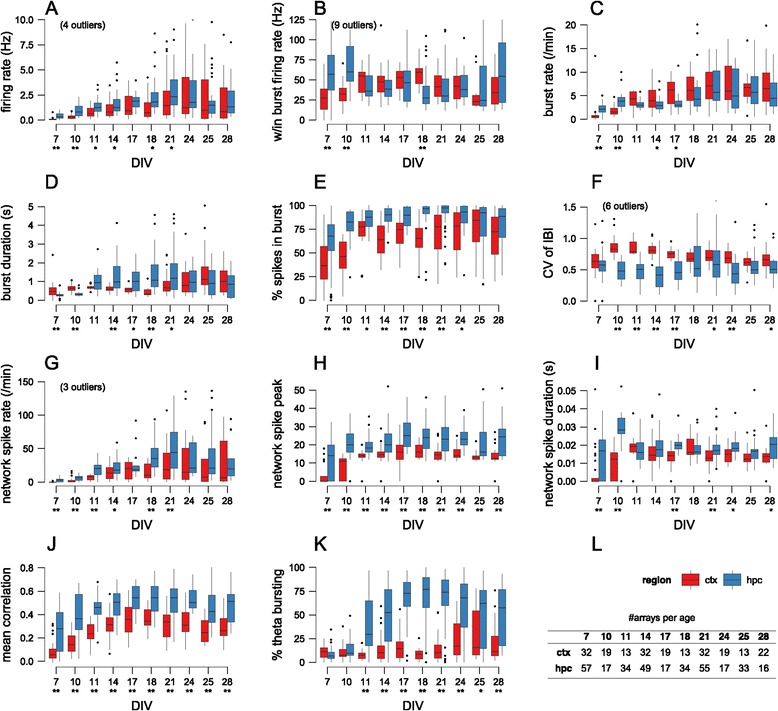
**Characterisation of spontaneous activity in hippocampal and cortical networks.** **(A–K)** Values of one feature (named on the *y*-axis) as a function of age. Box plots show the median and interquartile range, with whiskers extending out to the most extreme values within 1.5 times the interquartile range. Individual points outside this range are regarded as outliers and drawn as points; in a few cases these outliers are not drawn to keep the *y*-axis within a meaningful range. Underneath each age, stars denote significant difference of median values for cortical and hippocampal networks at either 0.05 (*) or 0.01 (**) level (Mann–Whitney test, with *P* values corrected for multiple comparisons with false discovery rate method). **(L)** Number of arrays analysed at each age. CTX, cortex; CV, coefficient of variation; DIV, days *in vitro*; HPC, hippocampus; IBI, interburst interval; w/in, within.

During development, there are slight, statistically significant differences in firing rates, with median firing rates being slightly higher for hippocampal networks, but overall there are no key differences at maturity (Figure 3A).

#### Bursting properties

Neurons typically fire in bursts, and are thought to be a reliable unit of neuronal information for functions such as coincidence detection and synaptic modification [12]. We find that bursts emerge around 7 DIV (days *in vitro*) and strengthen until about 14 DIV after which the bursting properties tend to stabilise. Among the bursting properties that we have measured, two factors seem to differentiate hippocampal and cortical networks. First, there is a higher fraction of spikes occurring within bursts for hippocampal networks (Figure 3E), although the difference is no longer significant by 28 DIV. Second, the spike trains from hippocampal networks seem to be more regular, as indicated by the lower coefficient of variation for interburst intervals (CV of IBIs) (Figure 3F). The other burst-based measures that we calculated, namely within-burst firing rate (Figure 3B), burst rate (Figure 3C) and duration (Figure 3D) show weaker differences between the two types of network.

#### Network activity

The previous measures analysed spiking data independently on each electrode. As a first approximation to assessing network activity, we used the concept of network spikes [13] to define the degree to which activity is coordinated across the entire array. At any time *t* we count the number of active electrodes; when this count exceeds a threshold, we say that a network spike has occurred. We measured three properties of these network spikes: their rate (per minute), their duration and their peak amplitude. Hippocampal networks tend to have more network spikes than cortical networks (Figure 3G) and the network spikes involve more electrodes across development (Figure 3H). The hippocampal network spikes tend to last slightly longer, although this is not consistent across development (Figure 3I). Overall, this suggests that network activity tends to be stronger and more coordinated in hippocampal than cortical networks.

#### Pairwise correlations

As a further method to detect coincident activity on electrodes, we computed correlation coefficients for all possible pairs of electrodes on the array. For any pair of spike trains, we computed the spike time tiling coefficient, as this measure is particularly well suited for relatively sparse spike trains [14]. For *N* (typically 59) electrodes on the array we compute *N*(*N*−1)/2 correlation coefficients (i.e. ignoring autocorrelations) and plot them as a function of the distance separating the two electrodes (Figure 2B). This technique has been used in studies of spontaneous activity in developing retina, and often reveals that correlations are dependent on distance, typically following a decaying-exponential profile [15]. However, we found that there is little, if any, distance dependence upon the correlation coefficients (Figure 2B), similar to that reported before [16]. We therefore decided to compute the average of all pairwise correlations.

Across all developmental ages, we find that the mean correlation is higher in hippocampal than in cortical networks (Figure 3J). From 7 DIV to 14 DIV, we see that the mean correlation becomes reliably stronger; after 14 DIV the correlations tend to stabilise.

#### Presence of theta bursting

The theta rhythm is a prominent 4 to 10 Hz oscillation measured in the hippocampus, and is thought to be involved in a range of neural functions [17]. We decided to examine whether our networks exhibited such oscillations by checking for peaks in the log interspike interval (ISI) histogram in the range 0.1 to 0.25 s. Figure 2B shows an example of one electrode (recording from a 14 DIV hippocampal network) that exhibited theta bursting. Our approach was to measure the fraction of electrodes exhibiting theta bursting. Perhaps the most striking feature that discriminates hippocampal from cortical networks is the presence of theta bursting in hippocampal networks. Although only about 10% of electrodes in hippocampal networks are classified as theta bursting at 7 DIV, after 11 DIV theta bursting is found on 50% to 75% of electrodes (Figure 3K). By contrast, most electrodes in cortical networks do not detect theta bursting, except at 25 DIV.

### Discrimination of hippocampal and cortical networks

Each of the 11 features documented in Figure 3 shows that there are significant differences between hippocampal and cortical networks. However, given that the distributions of values can overlap and yet still be statistically significant (e.g. firing rate at 21 DIV; Figure 3A), we cannot use individual features to reliably discriminate between the two types of network. We therefore used machine-learning techniques to address two related questions:

1. Out of the 11 features, which are the most important for discriminating hippocampal versus cortical networks?
2. Given a recording of a network at a given age, is it possible to predict the identity (hippocampal or cortical) of the network?

We therefore translated each 15-minute recording into an 11-dimensional vector, with element *i* of the vector storing the value of feature *i* measured from the recording. This vector is described below as a *feature vector* of the recording.

#### Principal component analysis

**Figure 4:**
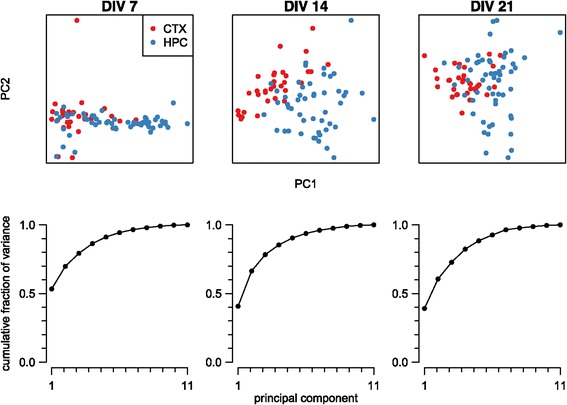
**Principal component analysis of hippocampal and cortical feature vectors.** Each column represents a principal component analysis of the 11-dimensional feature vectors of all recordings at a given age (days *in vitro*). In the scatter plot, each point represents one recording projected down into the two dimensions that account for maximal variance and is coloured according to its cell type. Each graph shows the cumulative fraction of variance accounted for by the principal components. CTX, cortex; DIV, days *in vitro*; HPC, hippocampus; PC, principal component.

If there is a consistent difference in the properties of hippocampal and cortical recordings, we would hope that the corresponding feature vectors cluster into two distinct regions. However, as these feature vectors are 11-dimensional, we must first reduce their dimensionality to visualise them. Many such dimensionality-reduction techniques are available; we chose to use the best-known method, principal component analysis. Figure 4 shows the projection of the feature vectors at three different ages down into two-dimensional space. At 7 DIV, there is significant overlap between the hippocampal and cortical recordings, which might suggest that it is hard to discriminate between the two types of recordings; however, at 14 and 21 DIV, the recordings from the same cell type cluster and there is significant separation of the hippocampal and cortical recordings. The graphs underneath each scatter plot show the cumulative percentage of variance accounted for by the components. In each case, the first two principal components account for at least 60% of the variance.

#### Classification of recordings

The principal component analysis suggests that, especially at the later ages, the feature vectors contain sufficient signal to discriminate between hippocampal and cortical networks. However, given the overlap between clusters, we next used classification techniques to quantify the degree to which the two classes of recording can be separated. We used two classification methods, detailed below. In both methods, two-thirds of the feature vectors at a given age are used to train a classifier to discriminate between the two types of recording. The remaining one-third of the feature vectors are then used as a test set to evaluate how well the classifier performs on data unseen during training.

We first used classification trees [18]. We built ten classifiers, one per age studied, to test whether the recordings could be grouped into cortical or hippocampal networks. We found that for any given age, the prediction accuracy of the trees was high – usually over 75% correct, depending on the age of the recording. (Performance would be 50% if there were no information to distinguish the two types of recording.) Once these trees had been built, we were able to interrogate them to find out which features were dominant in driving the classification of the network. At different ages, unsurprisingly, different features were dominant, but an overall trend clearly emerged when we averaged across developmental ages. Table 1 lists the features in decreasing order of their importance, along with their relative score (column 2).

**Table 1:** **Classifier performance at discriminating cortical from hippocampal cultures**

**Percentage correct at given age (DIV)**											
**Feature**	**Score**	**7**	**10**	**11**	**14**	**17**	**18**	**21**	**24**	**25**	**28**
CV of IBI	1.00	64	85	88	89	84	71	61	72	73	60
Theta burst	0.84	64	82	96	88	91	89	84	86	74	63
Mean correlation	0.50	67	89	96	91	86	94	89	84	75	75
Burst duration	0.33	72	86	97	91	92	90	89	82	77	83
Burst rate	0.32	78	84	97	92	90	95	89	91	74	78
% of spikes in bursts	0.25	82	86	92	94	92	94	89	87	77	79
Firing rate	0.21	83	84	94	94	90	94	92	89	74	78
NS peak	0.19	83	86	91	95	91	96	91	88	76	79
NS duration	0.19	83	89	90	92	89	93	93	86	72	78
Within-burst firing rate	0.16	83	89	96	93	92	96	93	87	77	79
NS rate	0.11	83	88	92	92	90	97	93	85	76	75

Features are listed in decreasing order of importance (score; column 2) normalised to the top score. The following numbers in each row *i* = 1, …, 11 are the mean percentage of correct classifications at each age using the top *i* features. CV, coefficient of variation; DIV, days *in vitro*; IBI, interburst interval; NS, network spike.

Out of the 11 features, three stood out. The most important was CV of IBI; this is a measure of firing regularity, which tends to be higher in hippocampal networks. Second, theta bursting is a key indicator (once it emerges at DIV 11) of hippocampal networks. Third, mean correlation is one of the top three features roughly half the time, and again is higher in hippocampal networks.

We chose classification trees as our first classification method primarily because of their simplicity (there are no free parameters) and ability to assess easily which features are driving the classification. Although their classification performance was good, we compared their performance against another common machine-learning technique, namely support vector machines (SVMs). We found that the SVM classifiers tended to result in slightly higher classification accuracy than the classification trees; e.g. when all 11 features were used, performance was 75% to 97% across ages (bottom row of Table 1).

Finally, given that our classifier trees provide us with a natural ordering of the importance of features, we asked how performance varied as we reduced the number of features that each recording is represented by. We found that performance remained high even as the number of features was gradually reduced (moving up through the rows of Table 1). It is clear, however, that multiple features are required for good classification; when only the single most important feature is used (top row of Table 1), performance was only just above chance at some ages. However, with only three or four features, we obtained good performance across all ages.

In conclusion, the results from the classifiers tell us that three features of network activity (CV of IBI, theta bursting and mean correlation) are strong predictors of whether a recording is from a hippocampal or a cortical network.

## Discussion

We have found that cultured networks of either hippocampal or cortical neurons generate spontaneous activity. These patterns of activity change during development and already by 7 DIV significant differences in their activity patterns begin to emerge. We have developed a quantitative framework for examining these activity patterns. By calculating 11 features of activity patterns, we can represent each recording of spontaneous activity as a point in (11-dimensional) feature space. When we examine recordings from any one given developmental age, we find that recordings from the same neuronal type (cortical or hippocampal) cluster in this feature space such that we can reliably discriminate between hippocampal and cortical networks.

Furthermore, out of the 11 features, we find that three are critically important for this classification in feature space. First, the CV of IBI was most important on average in driving the classification. Hippocampal spike trains tend to fire in bursts that are more regularly spaced than spike trains from cortical neurons. Second, after about 11 DIV, most electrodes in hippocampal recordings detect theta bursting, compared to a minority in cortical recordings. Third, the mean correlation between pairs of electrodes tends to be higher in hippocampal networks. These three measures are all relatively simple, and measure activity on either a single-electrode level or from pairs of electrodes. By contrast, although significant differences were found in the network spike measures, most importantly, the peak of network spikes (Figure 3F), these measures were not deemed to be critical in classification.

We have deliberately chosen simple features to characterise spiking activity to see if they suffice to discriminate between cortical and hippocampal networks. It is entirely likely that other more complicated measures of activity, particularly at the network level, may also reveal clear differences between these two types of network [10]. For example, network connectivity measures have been used to explore differences in spontaneous activity patterns between mature hippocampal and cortical networks in a range of frequency bands [19]. However, the simple measures we have chosen here suffice to differentiate the two types of network reliably. Likewise, even higher classification performance may be possible with more complex machine-learning techniques. However, our primary interest was to see whether in principle the feature space can be reliably separated with standard approaches [18]. Similar machine-learning methods are not yet routinely used in analysing spontaneous activity, although see [20] for a recent example showing how single-cell activity could be classified as either *in vivo* or *in vitro*. Finally, with the advent of a new generation of higher density MEAs containing up to 4,096 electrodes [21], it is likely that there are much richer patterns of activity than we describe here.

We believe that our framework lends itself nicely to many applications, for example in neurotoxicity testing where spontaneous activity from a network is recorded whilst it is exposed to a particular compound [7]. By building up a representative feature space of recordings from compounds known to be either toxic or safe, our approach can be used to predict the toxicity of novel compounds. This idea builds upon earlier work where mean profiles of activity in each condition were used as simple classifiers [22]. More recently, SVMs were used for toxicity prediction [23]. We imagine that our approach could also be used to detect the impact of particular genetic mutations, given earlier work suggesting that there may be significant differences in network activity [24].

### Limitations and future work

Our current work describes key quantitative differences in cortical and hippocampal spontaneous activity, but as yet we cannot explain what mechanisms might underlie these differences. One simple explanation might be that the two networks develop at different rates, and so comparing networks at the same age (measured as days *in vitro*) might be inappropriate. We also do not yet know whether the two types of network have different cellular compositions, such as the fraction of inhibitory interneurons or glia; even different fractions of GABA_A_ and GABA_B_ mediated inhibition can significantly regulate network states [25]. Alternatively, these differences in activity patterns could be driven by molecular differences in individual neurons and synapses, or by differences in network connectivity [10].

One potential concern about our current findings is that neuronal activity *in vitro* may provide little insight about neuronal activity *in vivo*. It is possible that other *in vitro* methods, such as brain slices, might produce more realistic activity patterns of spontaneous activity [26]. Recent machine-learning approaches have suggested, however, that cultured neurons generate spontaneous activity patterns more similar to *in vivo* activity than activity from organotypic slices [20]. However, we make no strong claims about whether our recordings from networks of cultured neurons can tell us about *in vivo* activity patterns; we believe that both cultured networks and slice preparations are only simple approximations to *in vivo* networks. Furthermore we believe the utility of *in vitro* approaches is that it we can readily study network properties under different environmental, genetic or pharmacological conditions.

## Conclusions

We report key differences in the developmental spontaneous activity patterns of cultured networks of hippocampal and cortical neurons. We have proposed a quantitative framework for evaluating these patterns. Our database of recordings and computer programs are all freely available for others to build upon. Future work in this area could be to dissect the cellular or network mechanisms driving the differences in network activity. For example, the differences between the cortical and hippocampus cultures could reflect molecular differences in cells or synapse or cellular differences in the populations of cells. Alternatively, differences in functional connectivity might partially account for these results [10]. Dissecting these differences will require a detailed understanding of the diversity of cell types defined by single-cell transcriptomes in these brain regions, which is still lacking.

## Methods

### Primary neuronal culture

Primary cultures of dissociated hippocampal and cortical neurons were prepared from mice at embryonic day (E) 17 to 18. Hippocampi and cortices were dissected from E17.5 mouse embryos (two to four, pooled) and transferred to papain (10 units/mL, Worthington, Lakewood, NJ, USA) for 22 min at 37°C. Cells were manually dispersed in Dulbecco’s modified Eagle’s medium containing 10% v/v foetal bovine serum and centrifuged twice at 400 *g* for 3.5 min. The final pellet was resuspended in Neurobasal/B27 supplemented with 0.5 mM Gln (Invitrogen, Paisley, UK), and dissociated cells (2×10^5^ per dish) were seeded in the centre of poly-D-lysine/laminin-coated MEAs (60MEA200/30-Ti, Multi Channel Systems, Reutlingen, Germany) containing 600 µl full Neurobasal medium. Zero-evaporation lids [27] were fitted and the MEAs housed in tissue culture incubators maintained humidified at 37°C and 5% CO _2_/95% air. Twenty-four hours post-plating, sample MEAs were placed on an inverted microscope with a heated stage (Axiovert 200; Zeiss, Cambridge, UK) and photographed through a 32 × phase objective at five different fields of view. These images were then analysed with CellProfiler [28] to quantify the neuronal density over the electrode array, giving an average value of 1,500 cells/mm ^2^.

At 3 to 4 days *in vitro*, cultures were fed by replacing 200 µl medium with pre-warmed fresh full Neurobasal medium. Cultures were subsequently fed using the same method after each recording, equating to a one-third medium change twice per week.

All procedures were performed in accordance with the United Kingdom Animals (Scientific Procedures) Act 1986. The mouse line used in this study was C57BL/6-*Tyr*^*c*-*Brd*^ (C57; albino C57BL/6).

### Multielectrode array recording

MEAs and all data acquisition hardware and software were from MultiChannel Systems (Reutlingen, Germany). Pairs of MEAs were interfaced with duplex 60 channel amplifiers and 15-minute recordings of spontaneous action potentials were made twice per week during the 4 weeks following plating. MEAs were heated and kept under a light flow of 5% CO _2_/95% air during recordings. Signals were digitised with a 128-channel analogue/digital converter card at a rate of 25 kHz and filtered (100 Hz high pass) to remove low-frequency events and baseline fluctuations. Action potentials were detected by the crossing of a threshold set to a fixed level of −20 µV, which typically approximated to 6 to 8 standard deviations from the baseline noise level. Record samples (1 ms pre- and 2 ms post-crossing of threshold) confirmed the characteristic action potential waveform. Application of tetrodotoxin (1 µM) totally abolished spiking activity, confirming the absence of false positive event detection using these methods. Spikes were not sorted to distinguish signals generated by individual neurons, so represent multiunit activity. Action potential timestamps were extracted using batch scripts written for NeuroExplorer [29] and analysed using software developed in the R statistical programming environment to compute parameters that quantitatively describe network activity. In total, 214 recordings were taken from 32 arrays of cultured cortical neurons, and 329 recordings from 61 arrays of cultured hippocampal neurons.

### Data analysis

To summarise a 15-minute recording of network activity, we computed the following features. As all recordings detected activity from multiple electrodes, we calculated summary scalar values (termed the array value below) by summarising the information from multiple electrodes. In this way, each recording was then represented as an 11-dimensional vector.

1. **Firing rate** The mean firing rate of each electrode was calculated. The array value was the median of all electrode firing rates.
2. **Within-burst firing rate** Bursts were detected independently on each electrode using our implementation of the max interval method from Neuroexplorer [29]. The parameters for burst detection are given in Table 2. For each electrode we calculated the mean of the firing rate during each burst. The array value was the median of the within-burst firing rates, ignoring electrodes where no bursts were detected.

**Table 2:** **Burst detection parameters**

**Parameter**	**Value**
Maximum beginning interspike interval	0.1 s
Maximum end interspike interval	0.25 s
Minimum interburst interval	0.8 s
Minimum burst duration	0.05 s
Minimum number of spikes in a burst	6
3. **Burst rate** For each electrode we calculated the number of bursts per minute. The array value was as per feature 2.
4. **Burst duration** The electrode value was the mean duration of bursts on that electrode. The array value was as per feature 2.
5. **Fraction of spikes in bursts** The electrode value was the total number of spikes classified as belonging to a burst divided by the total number of spikes on the electrode. The array value was as per feature 2.
6. **CV of IBI** The electrode value was the CV (standard deviation divided by mean) of the IBIs. The array value was as per feature 2.
7. **Rate of network spikes** Network spikes were defined as the array-wide average population activity [13]. It is defined by dividing time into small bins (here 3 ms) and counting the number of electrodes that generated at least one action potential in that bin. A network spike is then defined as the period when more than a threshold number of electrodes (here *n*=10) are simultaneously active. The array value was the number of network spikes per minute.
8. **Network spike peak** During each network spike we found the maximum number of active electrodes. The array value was the median of the values from each network spike in a recording.
9. **Network spike duration** The duration of each network spike was the time (in seconds) that the count of active electrodes exceeded the threshold value. The array value was as per feature 8.
10. **Mean correlation** Given two different spike trains from the recording, we calculated the correlation between them using the spike time tiling coefficient [14] with the coincidence window of *Δt*=5 ms. (We also tried *Δt*=50 ms and 0.5 ms, but results were qualitatively similar.) The array value was the mean of all distinct pairs of electrodes.
11. **Fraction of electrodes exhibiting theta bursting** For each electrode, the log ISI histogram was calculated and smoothed with the default kernel density estimation routine in R. A spike train was classed as showing theta bursting if a peak was present in the 4 to 10 Hz band of the histogram. The array value was the fraction of electrodes on the array that were classified as theta bursting.

The Mann–Whitney test was used to test whether the median array values at any given developmental age differed between the hippocampal and cortical networks. The *P* values were then corrected for multiple comparisons using the false discovery rate method [30].

### Clustering and classification

Standard principal component analysis was performed (with variance normalisation for each feature) for all feature vectors of any given age. Two standard machine-learning classifiers were tested: classification trees with boosting (random forests) and SVMs using radial kernel functions with *γ*=1/11 [18]. For each age, we built binary classifiers to predict the region (CTX/HPC) based upon the 11 features measured from each recording. For both classifiers, we used two-thirds of the recordings as training data, with the remaining one-third used as test data. Performance is reported as mean percentage of correct classifications, averaged over 500 repeats using different splits of the data into training and test sets. The classification tree approach allows us to assess the relative importance of features by measuring the degree to which they decrease the Gini index ([18], p. 319). These values were normalised to the value of the top-performing feature.

### Data and code availability

Statistical analysis was performed in the R programming environment using the SJEMEA package [31]. Data files containing the spike times from all recordings analysed here were stored in the HDF5 format using the framework created for spontaneous activity in retinal recordings [32]. The only addition to the framework was a new metadata item /meta/region containing either the phrase ‘CTX’ or ‘HPC’ depending on the network type. All data files and analysis code relating to this paper are freely available [33]. This includes all the material required to regenerate the figures and table in this article.

## Abbreviations

CTX: Cortex
CV: Coefficient of variation
DIV: Days *in vitro*
HPC: Hippocampus
IBI: Interburst interval
ISI: Interspike interval
MEA: Multielectrode array
NS: Network spike
SVM: Support vector machine
